# Prevalence of Hepatitis B and C virus infection and their co-relation with hematological and hepatic parameters in subjects undergoing Premarital Screening in the Jazan Region, Kingdom of Saudi Arabia

**DOI:** 10.12669/pjms.342.14278

**Published:** 2018

**Authors:** Saleh Mohammed Abdullah

**Affiliations:** Saleh Mohammed Abdullah, M.Sc., PhD, Department of Medical Laboratory Technology, Faculty of Applied Medical Sciences, Jazan University, Saudi Arabia

**Keywords:** HBV, HCV, Prevalence, Hematological, Hepatic, Parameter

## Abstract

**Objective:**

Hepatitis is a serious health concern with a high rate of mortality and morbidity world over. Saudi Arabia also has its course of the disease incidence. The data on the prevalence of the disease is still limiting. This study aimed to estimate the prevalence of hepatitis B virus [HBV] and hepatitis C virus [HCV] infection in the Jazan region and study its effects on hematological and hepatic parameters.

**Methods:**

This cross-sectional study was conducted at premarital screening centre located in King Fahd Central Hospital, Jazan, Kingdom of Saudi Arabia. A total of 7,826, Saudi couples undertaking premarital screening from Jazan region, were enrolled in the study and screened between January 2014 and June 2015 for hepatitis B virus and hepatitis C virus. Complete blood counts and hepatic profile were carried out for individuals who were Hepatitis B and or C virus positive.

**Results:**

A higher prevalence of hepatitis virus infection in male participants [HBV 1.9%; HCV 0.4%] than in females [HBV 1.43%; HCV 0.2%] was seen. The neutrophil-to-lymphocyte (NLR) and platelet-to-lymphocyte (PLR) ratios were significantly decreased among *HBV- and HCV-*infected patients. The concentration of hepatic enzymes showed a statistically significant increase in seropositive individuals. The levels of albumin were significantly decreased in individuals with hepatitis B and C when compared with the control group

**Conclusions:**

The study concludes that the prevalence of HBV infection among Saudi subjects in Jazan was higher than the prevalence of HCV infection, and both HBV and HCV were higher in men than in women.

## INTRODUCTION

Hepatitis is a critical global health issue that kills about 1.5 million people every year.^1^ It is an inflammatory disease of the liver that has a negative impact on the direct and indirect health costs in both developed and developing nations.^2^ Hepatitis can be either infectious or non-infectious in origin and amongst the infectious form of viral disease hepatitis is the most common one.^3^ Viral hepatitis is caused by five types of viruses A, B, C, D and E, of which only B and C may occur acutely or chronically and lead to chronic hepatitis.^4^ The most common form of viral hepatitis is Hepatitis B, and about 57 million persons are infected with the hepatitis B virus (HBV) world over.^5^ It is more prevalent in the Middle East as compared to America and Europe and ranges in prevalence from 0.6% in Iraq to more than 8% in Sudan.^1^ In the Eastern Mediterranean Region, the number of individuals infected with HBV is more than 21 million (3.3% of the population). Furthermore, it has been suggested that the prevalence of HBV could be higher among the general population than the proportion of blood donors.^5^

Hepatitis C affects about 3% of the world population; however, in developing nations of Africa, Asia and South America, it has a high prevalence of about 10%. Egypt also has a high prevalence of 20% due to an iatrogenic contamination.^4^

In 2007 Saudi Arabia ranked viral hepatitis as the second most common viral disease after chicken pox.^6^Earlier Saudi Arabia witnessed a high infection rate of hepatitis B with its prevalence being about 8.3%.^7^ In a community based epidemiological study Saudi children reported an HBsAg seroprevalence of about 7% and more than 70% prevalence of at least single HBV marker. These results were exigent and led to state and scientific intervention in the form of a strongly constituted universal HBV immunisation in the country, starting in 1989.^8^ To ensure success, this programme was succeeded by an additional program in 1990 for children at school entry, healthcare workers, and other high-risk groups. The immunisation programme was a success and resulted in the decrease of the occurrence of HBV from a hyperendemic disease to that of a low prevalence.^9^

In case of HCV, the data of its prevalence in KSA is limited as well as ambiguous. Screening centres for blood donors indicate HCV infection rates of 0.4-1.1% while premarital screening program estimates a prevalence of 0.33%.^10^-^14^ Recently a systemic review that included all published studies on the subject estimated that the prevalence ranges from 1.0-1.9%.^15^ These data suggest a need for substantial community-based prevalence studies to get an accurate incidence rate of the disease in Saudi Arabia. The lack of a confirmed data leads to impaired planning for the disease combat.^16^ Also, it is of significance to take in the account certain vulnerable populations that are at higher risk of developing the disease. E.g. HCV is more common in patients with the end-stage renal disease. 14 Although the immunisation programme along with education and improved living conditions of the people has led to a dramatic decline in the prevalence of HBV and HCV infection in Saudi Arabia, these viral diseases still cause significant morbidity and mortality, this has a negative impact on the country’s health care system and also leads to indirect economic losses.^9^ The present study was carried out in the Jazan region of Saudi Arabia, which holds a specific place in the country as it is the most densely populated region. Therefore the data is of considerable significance. This study aimed to determine the prevalence of hepatitis B and C virus infection among Saudi people undergoing premarital screening in the Jazan region and to study the effects of hepatitis B and C seropositivity on hematological and hepatic parameters.

## METHODS

### Source population

This cross-sectional study was conducted in 7,826 Saudi nationals from the Jazan region (3,913 males and 3,913 females, age:18-54) between January 2014 and June 2015 at the premarital screening centre, King Fahd Central Hospital, Jazan, Kingdom of Saudi Arabia. The study was approved by the Institutional Review Board of Jazan University (IRB No.102-17). The Review Board waived the requirement of an informed written consent for all individuals who voluntarily agreed to participate in this study, except in patients who tested positive for hepatitis B or C. The study was conducted in two phases, phase I where individuals visiting the premarital screening center were screened for hepatitis and phase II, where blood samples were collected from individuals who tested positive for hepatitis B or C and were ready to participate further in the study. Individuals visiting the blood bank to donate blood were recruited as controls (n=22) to compare their hematological and hepatic parameters with those of HBV and HCV seropositive individuals.

### Inclusion criteria for the study group

Individuals visiting the screening centre with no recent illness, no history of jaundice or hepatitis after the age of 10 years, and no recent history of medication intake, surgery or transfusion of blood or blood products.

### Inclusion criteria for control group

The control group was selected from voluntary blood donors visiting the blood bank. Blood donors were recruited after obtaining a detailed clinical history, physical examination and having the same inclusion criteria as the study group.

### Exclusion criteria

Individuals with a known history of hepatitis, other health issues, blood/blood component transfusions or major surgery in the previous six months were excluded.

### Blood sampling

Five ml whole blood samples were collected from all participants and divided into two parts: one part was transferred to a tube with Ethylenediamine tetraacetic acid (EDTA), while the other part was transferred to a plain tube and left undisturbed for 30-60 minutes. Serum was then collected after centrifugation of the blood and immediately stored at –20° C until analysis.

### Serological tests for HBV and HCV

HBV and HCV serological markers were evaluated using the ARCHITECT Immunoassay System (Abbott Laboratories, Wiesbaden, Germany) as per manufacturer’s guidelines and instructions. The HBsAg ULTRA kit (Bio-Rad, Marnes-la-Coquette, France) was used for the detection of HBsAg. Anti-hepatitis B core antibodies were detected with the anti-HBc PLUS kit (Bio-Rad, Marnes-la-Coquette, France). All of the anti-HBc positive samples were tested for anti-hepatitis B surface antibodies with the anti-HBs kit (Bio-Rad, Marnes-la-Coquette, France). The Murex anti-HCV kit (Murex Biotech Limited, Dartford, UK) was used for the detection of anti-HCV antibodies. Positive samples from the initial screening were retested to confirm the results.^17^

### Hematological and hepatic studies

Hematological and hepatic studies were carried out for the control group, and HBV and HCV positive individuals and complete blood counts were performed using an automated haematology analyzer, Sysmex Kx-21N (Tokyo, Japan). The neutrophil-to-lymphocyte ratio (NLR) and the platelet-to-lymphocyte ratio (PLR) were calculated. Liver function tests, including aspartate aminotransferase (AST), alanine aminotransferase (ALT), alkaline phosphatase (ALP), total bilirubin, direct bilirubin and albumin concentrations, were carried out by means of an automatic spectrophotometric technique, the Dimension RxL Max Clinical chemistry system (Siemens Healthcare Diagnostics Inc. Newark, DE, USA).

### Statistical analysis

Data analysis was performed using the Statistical Analysis System (SAS). The Student t test was used for comparison between seropositive and control groups. The results were considered to be statistically significant when *p* values were < 0.05. Comparison of hepatic parameters was made using one-way ANOVA with post-Hock Dunnett’s test.

## RESULTS

### Seroprevalence of HBV and HCV

The results showed that out of 3,913 Saudi males, 3,824 were seronegative, 74 tested positive for HBV and 15 were positive for HCV infection (Table-I). Conversely, out of 3,913 Saudi females, 3,851 were seronegative, while 56 tested positive for HBV and six were positive for HCV infection (Table-I). HBV seropositive male and female subjects were grouped based on their age in two categories, i.e., more than 30 years old and less than 30 years old. Out of 74 HBV positive male individuals, 55 were above the age of 30 years while 19 were less than 30 years old. In the female group, eight females were less than 30 years old, and 48 were more than 30 years old. HBV and HCV coinfection was not found in any individual enrolled in the study. The antibody test for hepatitis B virus-positive subjects showed that 5,557 individuals (72%) tested positive for anti-HBs antibodies, and 2,269 (28%) tested negative. Results from the anti-HBs and anti-HBc antibody tests in HBV seropositive individuals (total 130) are given in Table-II.

**Table-I:** Consolidated results of HBV and HCV positive male and female groups.

Total number of cases (7,826)		HBV				HCV			
Male	Female	Male		Female		Male		Female	
No.	No.	No.	%	No.	%	No.	%	No.	%
3913	3913	74	1.9	56	1.43	15	0.4	6	0.15

**Table-II:** Prevalence of anti-HBs and anti-HBc in male and female seropositive individuals.

Gender (n=130)	Anti-HBc		Anti-HBs	
	Positive	Negative	Positive	Negative
Male	63 (48.47%)	11 (8.47%)	60 (46.15%)	14 (10.77%)
Female	41 (31.53%)	15 (11.53%)	34 (26.15%)	22 (16.92%)
Total	104 (80%)	26 (20%)	94 (72.3%)	36 (27.69%)

Overall, a total of 151 individuals showed seropositivity for either HBV or HCV (130 positives for HBV and 21 for HCV). Only 55 subjects further participated in the second phase of the study; and provided blood samples for confirmation of the initial HBV and HCV results and to evaluate the hematological and hepatic parameters. Blood samples from the control group were collected in this phase of the study.

### Hematological findings

As shown in Table-III, haemoglobin levels and total leukocyte counts did not show any statistically significant differences between seropositive individuals and the control group. However, platelet and neutrophil counts were significantly reduced (*P* < 0.05) in HBV and HCV seropositive individuals. A statistically significant decrease (*P* < 0.05) was also noted in the NLR of HBV and HCV seropositive individuals compared with the control group (Table-III). The PLR was unaffected in HBV positive individuals, but this ratio was significantly decreased (*P* < 0.05) in HCV positive individuals (Table-III). The prothrombin time (INR) was also significantly prolonged in seropositive individuals, as shown in Table-III.

**Table-III:** Hematological parameters among the control group, HBV and HCV seropositive individuals.

Parameter/ Unit	Control	HBV	P	HCV	P
Hb (g/dl)	15.2± 4.1	13.5±3.9	0.651	13.2±3.08	0.716
WBCs (x10^9^/L)	6.4±2.7	5.97±2.02	0.092	5.85±1.67	0.089
Platelets (x10^9^/L)	245.5±6.14	186.4±5.98 *	0.001	165.75±7.02 *	0.001
Neutrophils (%)	63.4±10.4	48.6±5.5 *	0.02	40.9±9.3 *	0.021
Lymphocytes (%)	32.8±7.6	29.5±5.3	0.088	28.4±4.8	0.095
NLR	1.94±1.01	1.7±0.96 *	0.001	1.5±0.88 *	0.001
PLR	116.94	105.8	0.08	99.7*	0.041
Prothrombin time (INR)	0.98±0.32	2.1±1.04 *	0.003	1.73±0.79 *	0.001

Values are shown as Mean ± SD, Hb: Hemoglobin, WBC: Total white blood cell count,

NLR: Neutrophils-to-Lymphocytes ratio, PLR: Platelet-to-lymphocyte ratio.

### Hepatic findings

Table-IV shows the serum levels of AST, ALT, ALP, total bilirubin, direct bilirubin and albumin in the control group and individuals with HBV or HCV. The concentration of hepatic enzymes showed a statistically significant (*P* < 0.05) increase in seropositive individuals. Although total and direct bilirubin levels did not show any significant changes, a marginal increase in their levels was noted. The levels of albumin were significantly (*P* < 0.05) decreased in individuals with hepatitis B and C when compared with the control group.

**Table-IV:** Hepatic parameters among the control group, HBV and HCV seropositive individuals.

Parameter/ Unit	Control	HBV	P	HCV	P
AST IU/L	16.363±1.00	25.454±5.7*	0.02	30.454±5.7*	0.021
ALT IU/L	11.272±0.44	28.909±8.7*	0.005	38.636±8.7*	0.001
ALP IU/L	64.545±6.9	127.909±89.89	0.132	106.818±89.8*	0.001
TBIL µmol/L	14.100±0.88	14.836±7.48	0.664	16.618±7.48	0.564
DBIL µmol/L	0.527±0.17	3.563±3.04	0.199	4.800±3.04	0.151
ALB g/L	38.272±0.55	25.172±1.93*	0.01	23.090±1.93*	0.01

* Note: statistically significant; Values are shown as Mean ± SD

## DISCUSSION

Premarital disease screening is of considerable significance as it prevents a next-generation disease and can also provide an insight into the occurrence of certain diseases in the population.^18^ The mandatory premarital screening program in Saudi Arabia provides an excellent platform to estimate different disease prevalence in general population. This is of importance as it helps to obtain precise epidemiological data in general population than that in certain groups.^19^ The high rate of consanguineous marriages (exceeding 55%) in Saudi Arabia is a factor for high-risk births, especially hemoglobinopathies and hepatitis.^18^ With high incidences of hepatitis, it became highly significant to obtain datum on the prevalence of the disease. However, the studies are not still enough to obtain a comprehensive data. Our study was conducted in the most densely populated province of Saudi Arabia (120/km^2^ as of 2010) (https://en.wikipedia.org/wiki/Jizan_Region) and the study population were couples visiting the premarital screening clinic.

Our study revealed that 97.7% of screened male subjects were seronegative, while 1.9% and 0.4% were seropositive for HBV and HCV, respectively. In females, 98.41% were seronegative, while the prevalence of HBV and HCV was 1.43% and 0.15%, respectively. Local studies investigating this topic are very scant. A study conducted in 2009 showed that the prevalence of HBV in couples tested through the premarital screening program was 1.31%, while it was 0.33% for HCV.^14^ A study conducted in the Qassim region with couples planning to get married and above 20 years of age showed that the positivity for HBV to be 0.7% in 2008, 1.5% in 2009 and 2.04% in the year 2010. This study also reported that the prevalence of HCV to be 0.1% in 2008, 3% in 2009 and 0.83% in 2010.^20^ The results of these two studies are in close agreement with the results of the present study.

Compared with the general population and with blood donors, the prevalence values observed in this study were lower than those reported by previous studies involving different areas of the Kingdom of Saudi Arabia. The occurrence of HBV was reported to be 5.4% among Saudi blood donors in the Jazan region from June 1995 to June 1997.^21^ This prevalence was lower than that recorded during the years 1985 to 1996, which was 12%. A recent study also showed a lower incidence of HBV and HCV among blood donors in the Jazan region.^17^ This decline in the prevalence of HBV and HCV may be attributed to active vaccination programs and complementary immunological and therapeutic strategies that started in 1989. The present study also demonstrated that HBV is still the predominant type of hepatitis in Saudi Arabia, a result that is similar to previous studies.^22^ When the seropositive results were analyzed; it was observed that the incidence of HBV and HCV was higher in males than in females. This finding is consistent with a study conducted in the Riyadh region, where the prevalence was found to be 1.5% in females and 2.1% in males.^23^

Approximately 46.15% of male and 26.15% of female HBV seropositive individuals showed anti-HBs positivity in this study (Table-II). Contrary to a previous study which revealed a prevalence of anti-HBs antibodies of 47.6%, the higher prevalence of anti-HBs antibodies found in the current study could be explained by the active HBV immunization/vaccination program in the Saudi Arabia.^24^

When the anti-HBc results were analyzed, it was found that 11% of male and 15% of female participants tested positive for these antibodies. These findings are similar to those reported in another study, where anti-HBc antibodies were found in 8.9% of tested couples. 24

Hematological analysis of the HBV and HCV seropositive individuals showed a decrease in haemoglobin levels and total leukocytes that did not reach statistical significance, while a statistically significant decrease in platelet and neutrophil counts were seen. This decrease may be inversely proportional to the degree of hepatic damage, reflecting the involvement of the liver.^25^,^26^ The NLRs showed a statistically significant decrease in HBV and HCV positive individuals compared with controls (Table-II). This finding may reflect chronicity of the disease in some patients as suggested by a previous study finding that chronic hepatitis B patients with advanced fibrosis showed significantly reduced NLRs when compared with chronic hepatitis B patients with minimal or no fibrosis.^27^ Therefore, the NLR may be used as a noninvasive marker of fibrosis in patients with HBV.^28^ The PLR did not show a statistically significant reduction in HBV seropositive individuals. However, it was decreased in HCV positive individuals (Table-II). These findings are consistent with another study that reported a PLR value of 61±31 among HCV positive cirrhotic patients, to be significantly less than the control group (115±23).^29^ The prothrombin time (PT) and INR were also found to be increased in seropositive individuals when compared with the control group (Table-II). However, studies suggest that a prolonged PT and increased INR cannot be considered as an exact marker of cirrhosis.^30^

Biochemical analysis of the hepatic markers showed a statistically significant increase in the levels of AST, ALT, and ALP; these results are comparable to published data.^31^ Although the levels of ALT and AST in the present study were higher in HCV than in HBV seropositive individuals, the statistical analysis indicated this difference insignificant (Table-III). In contrast, ALP levels were higher in HBV than in HCV seropositive individuals. It has been observed that chronic hepatitis C is associated with variable ALT levels, ranging from normal to high. Persistently normal levels of ALT have been associated with a lower progression and occurrence of cirrhosis in patients with hepatitis.^32^

The level of ALP increases in liver diseases as a result of increased production and release into plasma, instead of impaired biliary secretion. The current study showed a considerable increase in ALP levels, validating previous notions concerning high levels of ALP in liver disease (Table-III). Total bilirubin and direct bilirubin levels were higher in both seropositive groups than in the control group, but the differences were not significant. In the present study, we also observed a significant reduction in the albumin levels. Albumin is one of the most essential proteins in plasma; it is synthesized by the liver and is an indicator of liver function. Liver disorders affect the synthesis of albumin since the liver is the only site of albumin synthesis.^33^

## CONCLUSIONS

Our study, therefore, presents the prevalence of the HBV and HCV in Jazan region for the couples seeking premarital screening and indicates a higher incidence of HBV than HCV with both being higher in men than in women. It is also indicative of the success of the immunization plan for hepatitis by the state authorities. The study, however, has a limitation that it lacked a follow-up of seropositive individuals for additional monitoring.

## References

[1] Habibzadeh F (2014). Viral hepatitis in the Middle East. Lancet.

[2] Udompap P, Kim D, Kim WR (2015). Current and future burden of chronic nonmalignant liver disease. Clin Gastroenterol Hepatol.

[3] (2016). World Health Organization. What is hepatitis?.

[4] Rutherford A, Dienstag JL, Greenberger NJ, Blumberg RS, Burakoff R, Viral hepatitis (2016). Current diagnosis & treatment:gastroenterology, hepatology, & endoscopy.

[5] (2017). World Health Organization. New hepatitis data highlight need for urgent global response.

[6] (2007). Ministry of Health of Saudi Arabia (MOH). A review of health situation. Annual Health Statistics Book.

[7] Al-Faleh FZ (1988). Hepatitis B infection in Saudi Arabia. Ann Saudi Med.

[8] Al Faleh F, Al Shehri S, Al Ansari S, Al Jeffri M, Al Mazrou Y, Shaffi A (2008). Changing patterns of hepatitis A prevalence within the Saudi population over the last 18 years. World J Gastroenterol.

[9] Abdo AA, Sanai FM, Al Faleh FZ (2012). Epidemiology of viral hepatitis in Saudi Arabia;Are we off the hook?. Saudi J Gastroenterol.

[10] Mehdi SR, Pophali A, Al-Abdul Rahim KA (2000). Prevalence of hepatitis B and C and blood donors. Saudi Med J.

[11] Bashawri LAM, Fawaz NA, Ahmad MS, Qadi AA, Almawi WY (2004). Prevalence of seromarkers of HBV and HCV among blood donors in eastern Saudi Arabia, 1998-2001. Clin Lab Haematol.

[12] El-Hazmi MM (2004). Prevalence of HBV, HCV, HIV-1, 2 and HTLV-I/II infections among blood donors in a teaching hospital in the Central region of Saudi Arabia. Saudi Med J.

[13] Madani TA (2007). Hepatitis C virus infections reported in Saudi Arabia over 11 years of surveillance. Ann Saudi Med.

[14] Alswaidi F, O'Brien S (2010). Is there a need to include HIV HBV and HCV viruses in the Saudi premarital screening program on the basis of their prevalence and transmission risk factors?. J Epidemiol Community Health.

[15] Sievert W, Altraif I, Razavi HA, Abdo A, Ahmed EA, Alomair A A systematic review of hepatitis C virus epidemiology in Asia, Australia, and Egypt. Liver Int.

[16] Alghamdi AS, Alqutub A, Abaalkhail F, Sanai FM, Alghamdi H, Altraif I (2015). SASLT position statement on the direct-acting antiviral agents for the treatment of hepatitis C virus infection. Saudi J Gastroenterol.

[17] Abdullah SM (2013). Prevalence of hepatitis B and C in donated blood from Jazan region of Saudi Arabia. Malays J Med Sci.

[18] Memish ZA, Saeedi MY (2011). Six-year outcome of the national premarital screening and genetic counseling program for sickle cell disease and β-thalassemia in Saudi Arabia. Ann Saudi Med.

[19] Karaosmanoglu HK, Aydin OA, Sandikci S, Yamanlar ER, Nazlican O (2012). Seroprevalence of hepatitis B:do blood donors represent the general population?. J Infect Dev Ctries.

[20] Aljarbou AN (2012). Current prevalence of HBV and HCV seropositivity:the initiative for attentiveness and deterrence of viral hepatitis in the Qassim region of Saudi Arabia. J Antivir Antiretrovir.

[21] Al-Tawfiq JA, Anani A (2008). Profile of viral hepatitis A, B and C in a Saudi Arabian hospital. MedSci Monit.

[22] Mansoor AA, Salih AI, Al-Jaroudi DH (2011). Screening of hepatitis B and C and human immunodeficiency virus in infertile couples in Saudi Arabia. Saudi Med J.

[23] Tripodi A, Mannucci PM (2011). The coagulopathy of chronic liver disease. N Engl J Med.

[24] Hayatbakhsh MM, Darvish Moghaddam S, Zahedi MJ, Shafiei M, Khalily Zade M, Assare M (2015). Seroprevalence of hepatitis B before marriage:A study on marriage candidates in the southeast of Iran;is it worthy of consideration?. Arch Iran Med.

[25] Witters P, Freson K, Verslype C, Peerlinck K, Hoylaerts M, Nevens F (2008). Review article:blood platelet number and function in chronic liver disease and cirrhosis. Aliment Pharmacol Ther.

[26] Ritto G, Bechlis Z, Stadlbauer V, Davies N, Francés R, Shah N (2011). Evidence of neutrophil functional defect despite inflammation in stable cirrhosis. J Hepatol.

[27] Kekilli M, Tanoglu A, Sakin YS, Kurt M, Ocal S, Bagci S (2015). Is the neutrophil to lymphocyte ratio associated with liver fibrosis in patients with chronic hepatitis B?. World J Gastroenterol.

[28] Meng X, Wei G, Chang Q, Peng R, Shi G, Zheng P (2016). The platelets-to-lymphocyte ratio, superior to neutrophils-to-lymphocyte ratio. correlates with hepatitis C virus infection. Int J Infect Dis.

[29] Parikh P, Ryan JD, Tsochatzis EA (2017). Fibrosis assessment in patients with chronic hepatitis B virus (HBV) infection. Ann Transl Med.

[30] Zainal IG, Safaa AA, Obead WK (2013). Biochemical parametersin relation to serum alpha feto proteinc &leptin levels in Iraqi pateints with chronic liver diseases. Int J Life Sci Farma Res.

[31] Yapali S, Talaat N, Lok AS (2014). Management of hepatitis B:our practice and how it relates to the guidelines. Clin Gastroenterol Hepatol.

[32] Woreta TA, Alqahtani SA (2014). Evaluation of abnormal liver tests. Med Clin North Am.

[33] Garcovich M, Zocco MA, Gasbarrini A (2009). Clinical use of albumin in hepatology. BloodTransfus.

